# Cartilage destruction in early rheumatoid arthritis patients correlates with CD21^−/low^ double-negative B cells

**DOI:** 10.1186/s13075-024-03264-2

**Published:** 2024-01-15

**Authors:** Katrin Thorarinsdottir, Sarah McGrath, Kristina Forslind, Monica Leu Agelii, Anna-Karin Hultgård Ekwall, Lennart T. H. Jacobsson, Anna Rudin, Inga-Lill Mårtensson, Inger Gjertsson

**Affiliations:** 1 Department of Rheumatology and Inflammation Research, University of Gothenburg, Box 480, 405 30 Gothenburg, Sweden; 2 Department of Rheumatology, Center for Rheumatology Research, University Hospital of Iceland, Reykjavík, Iceland; 3 Department of Immunology, University Hospital of Iceland, Reykjavík, Iceland; 4 Department of Clinical Sciences Lund, Section of Rheumatology, Lund University, Lund, Sweden; 5 Spenshult Research and Development Centre, Halmstad, Sweden; 6 Department of Rheumatology, Sahlgrenska University Hospital, Gothenburg, Sweden

**Keywords:** CD21^−/low^ DN B cells, Cartilage destruction, Early rheumatoid arthritis

## Abstract

**Background:**

Involvement of B cells in the pathogenesis of rheumatoid arthritis (RA) is supported by the presence of disease-specific autoantibodies and the efficacy of treatment directed against B cells. B cells that express low levels of or lack the B cell receptor (BCR) co-receptor CD21, CD21^−/low^ B cells, have been linked to autoimmune diseases, including RA. In this study, we characterized the CD21^+^ and CD21^−/low^ B cell subsets in newly diagnosed, early RA (eRA) patients and investigated whether any of the B cell subsets were associated with autoantibody status, disease activity and/or joint destruction.

**Methods:**

Seventy-six eRA patients and 28 age- and sex-matched healthy donors were recruited. Multiple clinical parameters were assessed, including disease activity and radiographic joint destruction. B cell subsets were analysed in peripheral blood (PB) and synovial fluid (SF) using flow cytometry.

**Results:**

Compared to healthy donors, the eRA patients displayed an elevated frequency of naïve CD21^+^ B cells in PB. Amongst memory B cells, eRA patients had lower frequencies of the CD21^+^CD27^+^ subsets and CD21^−/low^ CD27^+^IgD^+^ subset. The only B cell subset found to associate with clinical factors was the CD21^−/low^ double-negative (DN, CD27^−^IgD^−^) cell population, linked with the joint space narrowing score, i.e. cartilage destruction. Moreover, in SF from patients with established RA, the CD21^−/low^ DN B cells were expanded and these cells expressed receptor activator of the nuclear factor κB ligand (RANKL).

**Conclusions:**

Cartilage destruction in eRA patients was associated with an expanded proportion of CD21^−/low^ DN B cells in PB. The subset was also expanded in SF from established RA patients and expressed RANKL. Taken together, our results suggest a role for CD21^−/low^ DN in RA pathogenesis.

**Supplementary Information:**

The online version contains supplementary material available at 10.1186/s13075-024-03264-2.

## Introduction

Treatment with biologic drugs has for the last two decades transformed the prognosis of rheumatoid arthritis (RA), an autoimmune destructive joint disease. The B cell depleting therapy, rituximab, has been particularly successful in autoantibody positive RA patients, providing convincing affirmation of the involvement of B cells in RA pathogenesis [1]. The identification of the RA-associated autoantibodies, rheumatoid factor (RF) and anti-citrullinated protein antibodies (ACPA) has led to the discovery that these autoantibodies can be detected many years prior to clinical onset [2, 3]. Thus, the break of B cell tolerance occurs long before clinically detectable joint inflammation and raises the possibility that changes to the composition of a patient’s B cell pool could give vital clues to the nature of the approaching autoimmune pathogenesis at the point of diagnosis.

The division of B cells into phenotypic subsets using various cell surface markers commonly relies on IgD and CD27, where the latter is considered a marker for memory B cells (MBCs) and the presence or absence of IgD defines their status as unswitched or switched respectively [4]. Only a handful of studies have analysed the composition of the B cell population in the peripheral blood (PB) of early RA (eRA) patients, i.e. RA patients with disease duration less than 1 year according to EULAR definitions [5], and most report deficits relative to healthy donors (HDs) in both switched and unswitched MBCs [6–8]. There is also evidence that a decrease in CD27^+^ MBCs can be detected already a year prior to disease debut [9]. A CD21^−/low^ B cell population is expanded in chronic inflammatory states, e.g. in various autoimmune diseases such as Sjögren’s syndrome and systemic lupus erythematosus [10, 11] as well as chronic infectious diseases, e.g. HIV [12] and CVID [13]. However, the definition of the B cell population differs between these studies, which in part could be due to the studied diseases. We and others have shown that the CD21^−/low^ fraction of B cells and its subsets are expanded in patients with established (est) RA [8, 14–18], and in one of these studies, a positive correlation between disease activity and a CD21^−/low^ subset that was CD27^−^IgD^−^ (double negative, DN) CD11c^+^ was reported [18]. In our study on female patients with ACPA and/or RF positive estRA [16], we found the frequency of the CD21^−/low^ DN subset to be elevated relative to the same population in HDs. In addition, the frequency of CD21^−/low^ DN in PB also correlated with radiographic joint destruction suggesting a role in the pathogenesis. As for eRA, an increase in the frequency of CD21^−/low^ CD38^−^ subset has been described [8], whereas another study found no differences in the proportion of CD21^−/low^ CD11c^+^ in eRA compared to HD [19]. Thus, more information is needed on the composition of the B cell compartment in eRA and potential associations with clinical parameters such as disease activity and disease severity.

Here, we have analysed the associations between B cell subsets and clinical parameters including disease activity and joint damage in a large cohort of patients with eRA. Our aim was to provide insight into RA disease pathogenesis and possible therapeutic targets.

## Materials and methods

### Patients and healthy donors

Seventy-six patients with newly diagnosed RA, according to the American College of Rheumatology/European League Against Rheumatism 2010 criteria, were included in the study (Table 1). The patients who met the following inclusion criteria were eligible for the study: ≥ 18 years old, at least 2 swollen joints and 2 tender joints, RF-positive or ACPA-positive or C-reactive protein (CRP) ≥ 10 mg/L, moderate disease activity (> 3.2) by composite index Disease Activity Score28 (DAS28)-CRP, duration of symptoms (retrospective patient-reported pain in the joints) < 24 months, and no treatment with disease modifying anti-rheumatic drugs (DMARDs) or prednisolone. The patients were recruited at the rheumatology clinic at Sahlgrenska University Hospital in Gothenburg as well as the rheumatology clinic at the University Hospital at Malmö and Lund. Blood samples were taken from the patients within 1 week of diagnosis of RA. The patient group was compared to a group of twenty-eight age-and sex-matched controls HD (Table 1). The study was approved by the regional ethics committees of Gothenburg and Lund, Sweden, and all patients signed an informed consent form. Synovial fluid from patients with estRA (*n* = 5) was collected at the rheumatology clinic at Sahlgrenska University Hospital in Gothenburg. We also received information on age, sex and antibody status. Ethical permission did not allow for obtaining further clinical information.

**Table 1:** Baseline characteristics of patients with early and established RA and HD

**Characteristics**	**RA** ***N* = 76**	**HD** ***N* = 28**	**RA** ***N* = 32**	**RA** ***N* = 63**	**Est RA** ***N* = 5**
	All patients		Radiographs	Available CD27IgD data	SF
Female, *n* (%)	54 (71)	19 (68)	24 (75)	44 (70)	2 (40)
Age, year	54 (15)	55 (15)	55 (17)	52 (16)	62 (18)
Symptom duration (months)	6 (5)	NA	6 (4)	6 (5)	87 (74)^a^
Smoking, *n* (%)	11 (14)	NA	2 (6)	9 (14)	ND^b^
CDAI	28 (59)	NA	30 (14)	30 (12)	ND^b^
CRP, mg/L	24 (36)	NA	29 (44)	25 (39)	ND^b^
ESR, mm/h	33 (27)	NA	36 (29)	34 (29)	ND^b^
TJC68	19 (10)	NA	14 (10)	16 (10)	ND^b^
SJC66	16 (6)	NA	12 (7)	12 (6)	ND^b^
TJC28	9 (6)	NA	8 (7)	9 (7)	ND^b^
SJC28	9 (5)	NA	9 (5)	9 (5)	ND^b^
VAS-GH	58 (21)	NA	61 (20)	61 (20)	ND^b^
DAS28-ESR	5 (1)	NA	5 (1)	5 (1)	ND^b^
DAS28-CRP	5 (1)	NA	5 (1)	5 (1)	ND^b^
ACPA + , *n* (%)	63 (83)	NA	25 (78)	53 (84)	3 (75)^a^
RF + , *n* (%)	57 (75)	NA	22 (69)	47 (75)	2 (50)^a^
ACPA + RF + , *n* (%)	52(68)	NA	20 (63)	43 (68)	2 (50)^a^
ACPA − RF − , *n* (%)	8 (11)	NA	5 (16)	6 (10)	1 (25)^a^

Data are denoted as number of patients as well as proportions (%) for categorical data and means ± standard deviation for continuous data

*ACPA* anti-citrullinated protein antibody, *CDAI* Clinical Disease Activity Index, *CRP* C-reactive protein, *DAS28* Disease Activity Score 28 joints, *ESR* erythrocyte sedimentation rate, *NA* not applicable, *ND* not done, *RF* rheumatoid factor, *SF* synovial fluid, *SJC* swollen joint count, *TJC* tender joint count, *VAS-GH* visual analogue scale general health

^a^Two different ethical approvals were used to obtain synovial fluid, one of which did not allow clinical data to be analysed, so data were only available for 4 out of 5 patients

^b^Synovial fluid was obtained during an outpatient injection clinic and all joints were not assessed and laboratory data were not recorded

### Clinical disease assessments

Disease activity was evaluated by assessing the following parameters: Swollen Joint Count (SJC28), Tender Joint Count (TJC28), Disease Activity Score—Erythrocyte Sedimentation Rate (DAS28-ESR), DAS28-CRP, Clinical Disease Activity Index (CDAI), CRP and ESR. ACPA positivity was determined by a multiplexed anti-CCP test (BioPlex; Bio-Rad, Hercules, CA, USA), and RF positivity was determined by nephelometry (Beckman Coulter, Brea, CA, USA). Patients with ≥ 20 IU/ml ACPAs or RF in the serum were considered ACPA or RF positive, respectively.

For thirty-two patients radiographs of hands and feet were taken at the time of diagnosis and evaluated by a certified assessor, blinded to clinical data, autoantibody and B cell status. The modified Sharp van der Heijde score (mSHS, 0–448), including 16 areas for erosions and 15 areas for joint space narrowing in each hand and 6 areas (for both erosions and joint space narrowing) in each foot, was used [20].

### Flow cytometry

Peripheral blood and synovial fluid mononuclear cells (PBMCs and SFMCs) were isolated from whole blood and synovial fluid, respectively, using Lymphoprep (Axis-Shield, Oslo, Norway). SFMCs were frozen in 10% DMSO FBS and stored at − 150°C for between 13 and 30 months. SFMCs were thawed and washed with PBS. Fc receptors were blocked with 1% mouse serum (in-house) for 15 min on ice for both SFMCs and PBMCs and subsequently surface stained at a final concentration of 0.5–1 × 10^6^ in 100 μl for 30 min on ice. Antibodies and dilutions used in flow cytometry analysis as shown in Supplementary Table 1. Cells were acquired on a FACSCanto2 and BD FACSLyric. Data were analysed using the Flow Jo software (Tree Star Ashland, OR, USA).

### Gating strategy for flow cytometry

**Fig. 1:**
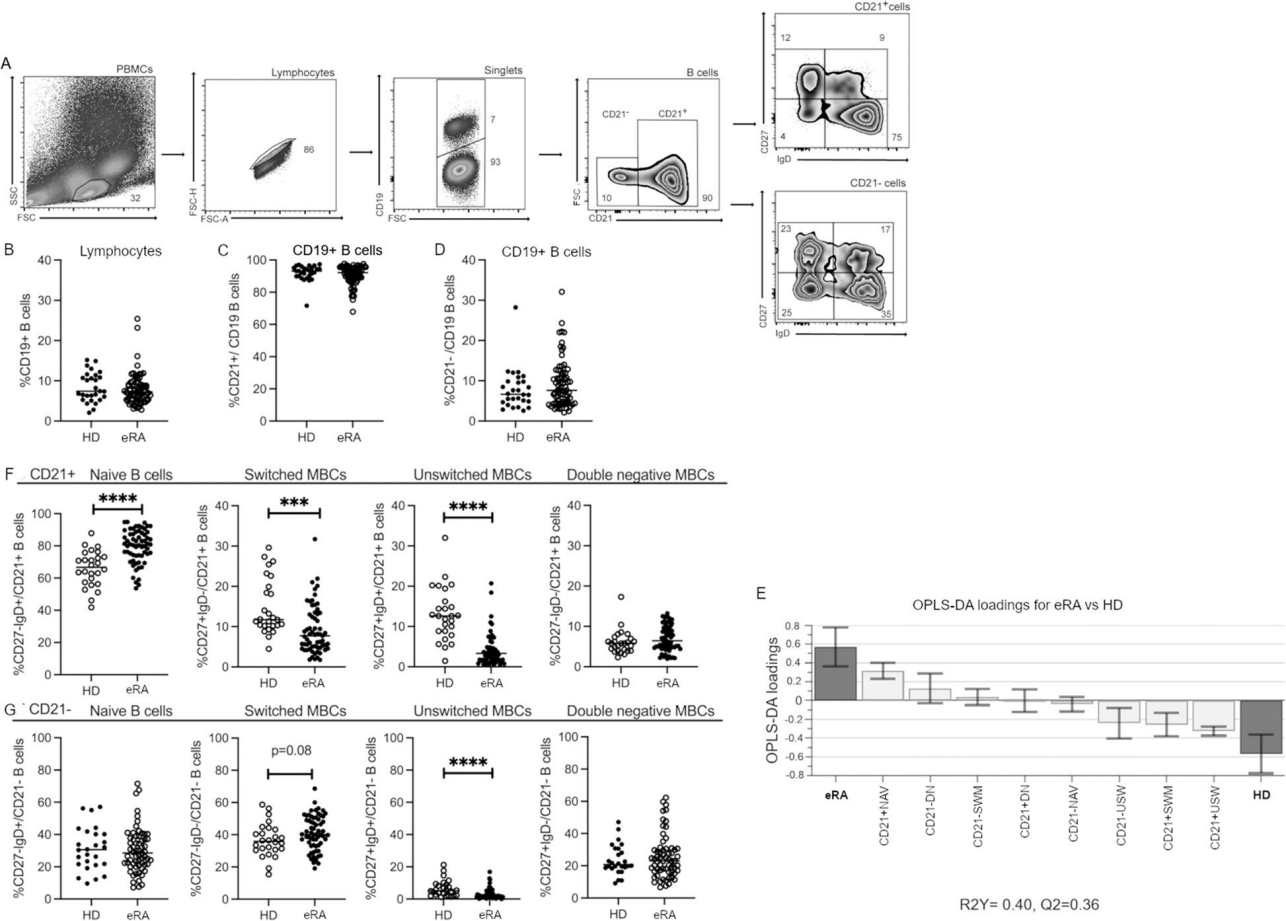
Proportions of B cells and B cell subsets in patients with eRA and HD and the association between subsets and disease. **A** Gating strategy showing gating on PBMCs for lymphocytes, which were further gated for singlets and thereafter CD19^+^ B cells. CD19^+^ B cells were further divided into CD21^+^ and CD21^−^ cells. The CD21^+^ and CD21^−^ were gated on using a CD27 vs IgD strategy. **B** Proportion of CD19^+^ B cells of peripheral blood lymphocytes. **C** Proportion of CD21^+^ cells of CD19 ^+^ B cells. **D** Proportion of CD21^−^ cells of CD19 ^+^B cells. **E** Multivariate factor analysis: An OPLS-DA loading plot showing the association between the different B cell subsets (*X*-variables) and eRA patients and HD, respectively (*Y*-variables). *X*-variables represented by bars pointing in the same direction as a *Y*-variable are positively associated with that *Y*-variable, whereas *X*-variables pointing the opposite direction are negatively related to that *Y*-variable. R2Y indicates how well the variation of *Y* is explained and Q2 how well *Y* can be predicted. **F** The proportion of the following B cell subsets: Naïve and transitional cells (NAV, CD27^−^IgD^+^), switched memory cells (SWM; CD27^+^IgD^−^), unswitched memory cells (USW, CD27^+^IgD^+^) and double-negative memory cells (DN, CD27^−^IgD^−^) of parent population CD21^+^ B cells. **G** The proportion of the following B cell subsets: naïve and transitional cells (NAV, CD27^−^IgD^+^), switched memory cells (SWM; CD27^+^IgD^−^), unswitched memory cells (USW, CD27^+^IgD^+^ and double-negative memory cells (DN, CD27^−^IgD^−^) of parent population CD21^−/low^ B cells. Mann–Whitney *U* test was used for statistical comparisons. *** *p* < 0.001, ****p < 0.0001. Between 1.5 × 10^5^ and 7 × 10^6^ events were recorded per sample. HD, healthy donors; eRA, early rheumatoid arthritis

B cells were identified in PBMCs or SFMCs as single lymphocytes expressing CD19, which were then divided according to their expression of the CD21 coreceptor into CD21^+^ and CD21^−^ populations (Fig. 1A). By gating for CD27 vs IgD, we defined four B cell subsets: naive and transitional cells (NAV, CD27^−^IgD^+^), switched MBCs (SWM, CD27^+^IgD^−^), unswitched MBCs (USW, CD27^+^IgD^+^) and double-negative MBCs (DN, CD27^−^IgD^−^); these populations were identifiable in both CD21^+^ and CD21^−^ populations.

### Statistical analysis

The relation between eRA and healthy donors or between clinical B cell subset proportions and clinical parameters was assessed by means of multivariate factor analysis**.** Two-class discriminant analysis (OPLS-DA) was used to examine whether eRA could be discriminated from the healthy donors based on the various/different B cell subset proportions. Data were normalized using a log transformation and were further scaled to unit variance (by dividing each variable by its standard deviation) so that all the variables were given an equal weight regardless of their absolute value. The loading vectors were normalized to length 1. OPLS model performance was assessed according to R2 (amount of variation explained) and Q2 (how well the outcome can be predicted by the model in a cross-validation sample). Aforementioned statistical analyses were conducted in SIMCA version 17.0.1; Umetrics, Umea, Sweden. B cell populations between two groups were compared according to either paired *T* test or Mann–Whitney tests, and for ≥ 2 groups, Kruskal–Wallis test or Friedman test with Dunn’s multiple comparisons was used. Associations between CD21^−/low^ DN and radiological measures, i.e. mSHS, joint space narrowing score (JSN) and erosion score (ES), were examined in linear regression models that were adjusted for age, sex, autoantibody status and smoking, if significant. Statistical analyses were conducted with SAS 9.4 (SAS Institute Inc., Cary, NC, USA). *p*-values < 0.05 were considered statistically significant.

## Results

### Characteristics of the study population

Seventy-six eRA patients and 28 age-and sex matched HD were included in the study. The participant demographics are summarized in Table 1. Both eRA patients and HD were mostly women, 71% and 68%, respectively. The mean tender joint count 28 (TJC28) was 9 (± 6), and the mean swollen joint count 28 (SJC28) was 9 (± 5). Similarly, the cohort had a moderate disease activity score 28-CRP (DAS28-CRP) of 5.0. The majority of eRA patients were ACPA and/or RF positive (68%). Only 14% of the eRA patients were smokers. Around 59% of eRA patients had bone erosions and 63% cartilage loss on radiographs of hands and feet taken at the time of diagnosis.

### The B cell compartment in eRA patients is disturbed compared to controls

First, we asked whether the B cell compartment in eRA patients differed from that in HD. To do so, flow cytometric evaluation of the B cell compartment was conducted whereby B cells (CD19^+^) were divided into CD21 + and CD21^−/low^ populations, and using the phenotypic markers CD27 and IgD, these were further divided into the following established cell subsets, i.e. switched memory (SWM, CD27^+^IgD^−^), unswitched memory (USW, CD27^+^IgD^+^), naïve and transitional (NAV, CD27^−^IgD^+^) and double-negative cells (DN, CD27^−^IgD^−^) (Fig. 1A). The frequency of total B cells (CD19^+^ cells) was similar in eRA patients and HD (Fig. 1B) as were the frequencies of the total CD21^+^ and CD21^−/low^ populations (Fig. 1C and D). OPLS-DA was used to determine whether any subsets of these B cell populations associated with eRA. The B cell subsets that showed the strongest association with eRA (positive or negative) are displayed in the column plot in (Fig. 1E).

The CD21^+^ cell subsets with the strongest positive associations to eRA were the NAV cells, and those with the strongest negative association were the USW and SWM cells (Fig. 1E). This was confirmed with univariate analyses, which demonstrated that the frequency of CD21^+^NAV cells was increased and the frequency of CD21^+^USW and CD21^+^SWM cells decreased in eRA compared to HD (Fig. 1F).

Looking at the CD21^−/low^ subsets in the OPLS-DA, we found that DN cells had the strongest positive and the USW cells the strongest negative association to eRA (Fig. 1E). Further univariate analysis did not reach significance for the CD21^−/low^ DN cells but could confirm the CD21^−/low^ USW association, i.e. that the frequency of CD21^−/low^ USW was significantly decreased in eRA patients compared to HD (Fig. 1G).

### Frequency of CD21^−/low^ DN cells correlates with joint space narrowing in eRA

**Fig. 2:**
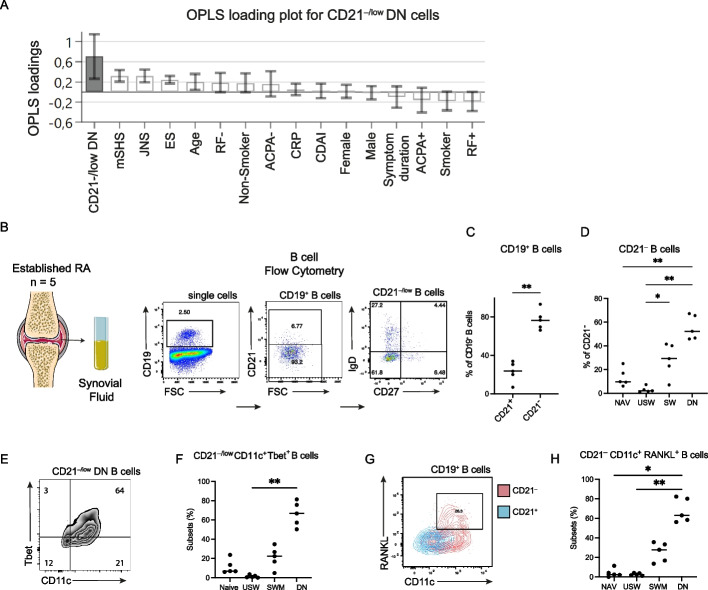
Association between CD21^−/low^ double-negative memory B cells and joint space narrowing score. **A** OPLS analysis depicting the association between the frequency of CD21^−/low^ double-negative memory cells (DN, CD27^−^IgD^−^) and clinical factors in eRA patients. **B** SF from swollen knees of rheumatoid arthritis patients (*n* = 5) was collected. Sample handling, staining and gating strategy as described in methods section. CD21^−/low^ B cells are increased in SF of estRA patients (**C**) and are mainly DN (**D**). The majority of the CD21^−/low^ DN are CD11c^+^Tbet^+^ (**E**) and the majority of the CD21^−^CD11c^+^Tbet^+^ are DN (**F**).** G** A representative FACS plot showing that RANKL^+^CD21^−/low^ B cells co-express CD11c. **H** The majority of CD21^−/low^ CD11c^+^RANKL^+^ cells are DN. B cell populations between two groups were compared using paired *T* test and Friedman test with Dunn’s multiple comparisons. *p < 0.05, ** *p* < 0.01. Between 2 × 10^5^ and 5.4 × 10^5^ events were recorded per sample. estRA, established rheumatoid arthritis; SF, synovial fluid; eRA, early rheumatoid arthritis

Next, we asked whether any of these aforementioned B cell subsets were associated with eRA clinical features, i.e. joint destruction, disease activity and autoantibody status as well as age and sex. We have previously shown that the frequency of CD21^−/low^ DN cells correlated with joint damage in estRA, and our objective was to investigate whether we could detect a similar relationship in eRA. The OPLS model displayed associations for CD21^−/low^ DN cells and various clinical factors (Fig. 2A). Total mSHS, consisting of joint space narrowing score (JSN) and erosion score (ES), was positively associated with CD21^−/low^ DN cells, after adjusting for age (Table 2). Notably, the frequency of CD21^−/low^ DN was significantly associated with JSN (*p* = 0.03), linking CD21^−/low^ DN cells with joint damage, in eRA (Table 2). Sex was neither associated with CD21^−/low^ DN cells nor mSHS and its composites. We did not find any association between APCA or RF titres and measures of joint destruction, i.e. ES, JSN and mSHS, indicating that the autoantibodies did not have a confounding effect on the association between CD21^−/low^DN cells and mSHS as well as JSN. This supports the hypothesis that ACPA and RF are not influencing the CD21^−/low^ DN association with joint destruction. The OPLS-DA models for the CD21^+^ cell subsets, i.e. CD21^+^ NAV, SWM and USW and for the CD21^− ^USW subset, did not show any association with clinical factors.

**Table 2:** Associations between mSHS, joint space narrowing score, erosion score and frequency of CD21^−/low^ double-negative B cells as well as age

Dependent variable	Predictors	Parameter estimate (95% CI)	*p*-value
**mSHS**^**a**^	Intercept	− 28.55 (− .44.34; − 12.76)	0.0009
	CD21^- ^DN cells	0.30 (− 0.03; 0.62)	**0.07**
	Age	0.50 (0.28; 0.72)	** < 0.0001**
**JSN**^**b**^	Intercept	− 21.88 (− 34.45; − 9.31)	0.0013
	CD21^- ^DN cells	0.29 (0.03; 0.54)	**0.03**
	Age	0.31 (0.13; 0.48)	**0.001**
**ES**^**c**^	Intercept	− 6.35 (− 11.76; − 0.94)	0.02
	CD21^- ^DN cells	− 0.02 (− 0.13; 0.09)	0.71
	Age	0.20 (0.13; 0.28)	** < 0.001**
	Smoking	5.13 (0.22; 10.04)	**0.04**

*JSN* joint space narrowing score, *ES* erosion score, *mSHS* modified Sharp van der Heijde score

^a^mSHS model: adjusted R2 = 51%, i.e. 51% of the variation in mSHS is explained by the B cell population and age

^b^JNS model: adjusted R2 = 20%, i.e. CD21-DN cells explain 20% of the variation in JNS

^c^ES model: adjusted R2 = 46%, i.e. age and smoking explain 46% of the variation in ES

Taken together, our results suggest that CD21^−/low^ DN cells are involved in joint destruction in eRA patients.

### CD21^−/low^ DN cells in synovial fluid co-express RANKL and CD11c

To investigate whether CD21^−/low^ DN cells had the potential to directly drive the mechanism of joint damage, SF from the inflamed joints of estRA patients was assessed for the presence of CD21^−/low^ DN cells and their phenotype (Fig. 2B). This included the surface expression of receptor activator of the nuclear factor κB ligand (RANKL), which is known to drive osteoclastogenesis and contribute to joint destruction. We confirmed previous results that B cells in SF were predominantly CD21^−/low^ (Fig. 2C) [16, 21]. Around 50% of the CD21^−/low^ cells were DN and 30% SWM (Fig. 2D). Further exploration of the CD21^−/low^ DN cells revealed that they largely expressed the integrin CD11c and transcription factor Tbet with the majority of CD21^−/low^ CD11c^+^ Tbet^+^ B cells coming from the DN compartment (Fig. 2E, F). Moreover, RANKL^+^ B cells were exclusively CD21^−/low^ and could be further characterized by the co-expression of the integrin CD11c (Fig. 2G). Furthermore, 60% of CD21^−/low^ CD11c^+^RANKL^+^ cells were from the DN subset, while SWM contributed only to 30% of RANKL and CD11c expressing SF B cells (Fig. 2H).

In conclusion, our analyses revealed an altered B cell compartment in eRA with CD21^−/low^ DN cells linked to joint space narrowing and an expansion of CD21^−/low^ DN cells in SF from the inflamed estRA joint where they expressed CD11c and Tbet. This SF B cell subset was further characterized by the expression of RANKL, demonstrating a capacity to promote osteoclastogenesis and revealing a possible role in the pathogenesis of RA.

## Discussion

In this study, the enrolment of untreated eRA patients aimed to limit the confounding effects of chronic inflammation and immune suppressive treatments on the B cell compartment and to provide an accurate and robust analysis of the B cell population in the early phase of RA. We have analysed the main B cell subsets in PB from eRA patients in which we can demonstrate a significant association between the frequencies of the CD21^−/low^ DN MBCs and joint destruction. Amongst the relatively few studies regarding B cell subsets in patients with eRA, our current report is unique as it separates the B cell populations according to expression levels of the complement 2 receptor, CD21. Lack of or low levels of CD21 on B cells are characteristic of pathologies related to some chronic infections and autoimmunity [22]. We have both identified changes in the composition of the B cell population and additionally analysed those in relation to clinical outcomes.

CD21^−/low^ B cell subsets have been linked to autoimmune diseases [10, 11, 14], chronic infections [12, 23–25] and immunodeficiency [13, 14] and comprise around 5% of the B cell population in healthy donors [26] and around 10% in seropositive estRA [16]. Our finding that the CD21^+^ B cell population contains a lower frequency of SWM and USW in eRA patients than in HD is in concordance with previous works [6–9]. Moreover, in agreement with others studying estRA [27–29], we did not find any differences between eRA patients and HD in the frequency of the CD21^+^ DN subset; this outcome notwithstanding, there are reports in both eRA [30] and estRA [30–32] of increases in the frequency of CD19^+^ DN.

In a previous study, a negative correlation between female sex and MBCs as well as between age and MBCs, transitional B cells and plasmablasts was described, but no association with the CD21^−/low^ subset was shown [8]. We found no differences between gender and any of the analysed B cell subsets, but there is a positive association between CD21^−/low^ DN cells and age in the current study. The frequency of DN cells as a proportion of the total B cells has been linked to ageing; one study in which age correlated positively with the CD19^+^DN subset [33] and a second in which the CD21^−/low^ CD27^+^ B cell subset correlated positively with age in older women with RA [34]. Clearly, there is scope for further investigation of the relationship between different B cell subsets with age, in both health and disease.

We did not find any correlations between disease activity and any particular B cell subset. Different studies have reported varying findings regarding B cell associations with DAS28. Studies have found a positive correlation with CD21^−^DN CD11c^+^ B cells [18], CD86^+^ B cells [7] and plasmablast frequencies in estRA and treated early RA [8, 32]. Additionally, opinion is divided on whether or not the frequencies of CD19^+^ DN cell subsets from patients with estRA correlate with DAS28 [28, 31].

We have previously shown in estRA that the frequency of CD21^−/low^ DN cells correlated significantly with joint destruction as measured by mSHS [16]. In the present study, it is clear that also in eRA patients with very little (or no) joint damage, CD21^−/low^ DN still associate with cartilage destruction. The implication of a specific B cell subset in the disease pathology at this point offers a compelling direction for further studies. In this study, the CD21^−/low^ DN population dominated the B cell population in the SF of estRA patients. Other studies have in addition to the DN subset also shown SWM to be a significant component of the SF B cell compartment [35, 36]. CD21^−/low^ B cell subset in different chronic autoimmune and inflammatory conditions have been described using diverse markers, but a common characteristic of these subsets is the expression of CD11c and Tbet [22]. Indeed, our CD21^−/low^ DN SF subset is mainly CD11c^+^Tbet^+^. It has been shown that CD21^−^DN CD11c^+^ cells are able to activate fibroblast-like synoviocytes (FLS), which in turn produce matrix metalloproteinases (MMPs) and IL-6, facilitating cartilage destruction [18]. Of further interest, we found that the SF CD21^−/low^ DN CD11c^+^Tbet^+^ subset was largely RANKL^+^, which suggests an additional role in bone destruction, as RANKL promotes osteoclastogenesis. This is supported by previous results that found FcRL4^+^ B cells in SF of estRA patients were also CD21^−/low^, RANKL^+^ and largely of the DN phenotype [35]. The association of CD21^−/low^ DN cells and joint damage both at early and later stages of RA and their expansion in the RA joints suggests that these cells are pathogenic and provide a possible treatment target.

## Conclusions

A direct role for CD21^−/low^ DN B cells in the destructive process in the inflamed joint implied by these results is of particular interest in the context of the pathogenesis and treatment of RA.

### Supplementary Information

**Additional file 1: Table S1.** Antibodies used for flow cytometry.

**Additional file 2: Table S2. **Different medication used by eRA patients included in study.

**Additional file 3: Table S3. **Comorbidities of eRA patients included in study.

## Data Availability

The datasets used during the current study are available from the corresponding author on reasonable request.

## Abbreviations

ACPA: Anti-citrullinated protein antibody
BCR: B cell receptor
CDAI: Clinical Disease Activity Index
CRP: C-reactive protein
DAS28: Disease Activity Score, including a 28-joint count
DMARD: Disease modifying anti-rheumatic drug
DN: Double negative
eRA: Early rheumatoid arthritis
ES: Erosion score
ESR: Erythrocyte sedimentation rate
estRA: Established rheumatoid arthritis
FLS: Fibroblast-like synoviocytes
HD: Healthy donor
Ig: Immunoglobulin
JSN: Joint space narrowing score
NAV: Naive and transitional B cells
MBC: Memory B cells
MMP: Matrix metalloproteinase
mSHS: Modified Sharp van der Heijde score
N/A: Not applicable
OPLS-DA: Orthogonal projections to latent structures discriminant analysis
PB: Peripheral blood
PBMC: Peripheral blood mononuclear cells
RA: Rheumatoid arthritis
RANKL: Receptor activator of the nuclear factor κB ligand
RF: Rheumatoid factor
SF: Synovial fluid
SFMC: Synovial fluid mononuclear cells
SJC: Swollen joint count
SWM: Switched memory B cells
TJC: Tender joint count
USW: Unswitched memory B cells
VAS-GH: Visual analogue scale general health
