# Supplementary material for: Cartilage destruction in early rheumatoid arthritis patients correlates with CD21−/low double-negative B cells
Source: Arthritis Res Ther. 2024 Jan 15;26:23. doi: 10.1186/s13075-024-03264-2 (PMC10789032; doi:10.1186/s13075-024-03264-2)
Supplement: Supplementary file 1 — Additional file 1: Table S1. Antibodies used for flow cytometry. [file 13075_2024_3264_MOESM1_ESM.docx]

**Table S1. Antibodies used for flow cytometry**

| **Antibody Clone** | **Company** | **Dilution** |
| --- | --- | --- |
| CD19-V500 HIB19 | BD Horizon | 40 |
| CD19-BV510 HIB19 | Biolegend | 40 |
| CD21-Pe-Cy5 B-ly4 | BD Pharmingen | 40 |
| CD21-PerCP Cy5.5 Bu32 | Biolegend | 50 |
| CD23-APC M-L233 | BD Pharmingen | 80 |
| CD24-PE-Cy7 ML5 | BD Pharmingen | 6 |
| CD27-PE L128 | BD | 40 |
| CD27-PE Cy7 M-T271 | BD Pharmingen | 50 |
| CD27-BV421 M-T271 | BD Horizon | 20 |
| CD38-BV421 HIT2 | BD Horizon | 6 |
| IgD-PE IA6-2 | BD Pharmingen | 40 |
| IgD-APC H7 IA6-2 | BD Pharmingen | 50 |
| IgD-V500 IA6-2 | BD Pharmingen | 100 |
| CD11c-FITC B-ly6 | BD Pharmingen | 20 |
| Tbet-PE 4B10 | Invitrogen | 40 |
| RANKL-APC MIH24 | Biolegend | 25 |
