# Supplementary material for: Cartilage destruction in early rheumatoid arthritis patients correlates with CD21−/low double-negative B cells
Source: Arthritis Res Ther. 2024 Jan 15;26:23. doi: 10.1186/s13075-024-03264-2 (PMC10789032; doi:10.1186/s13075-024-03264-2)
Supplement: Supplementary file 2 — Additional file 2: Table S2. Different medication used by eRA patients included in study. [file 13075_2024_3264_MOESM2_ESM.docx]

**Table S2. Different medication used by eRA patients included in study**

| **Medication** | **eRA**  **(N=59)** |
| --- | --- |
|  | *Number (%)* |
| None | 3 (5) |
| NSAIDs | 49 (83) |
| Paracetamol | 21 (36) |
| Proton pump inhibitors | 10 (32) |
| Bisphosphonates | 6 (10) |
| Calcium carbonate, cholecalciferol | 9 (15) |
| Levothyroxine | 6 (10) |
| Acetylsalicylic acid | 6 (10) |
| Beta-blockers | 5 (8) |
| ACE inhibitors /ARBs | 11 (19) |
| Calcium channel blockers | 8 (14) |
| Diuretics | 6 (10) |
| Insulin | 3 (5) |
| Metformin | 2 (3) |
| Statins | 5 (8) |

Data are number of patients (%) for categorical data.
ACE inhibitors, Angiotensin-converting enzyme inhibitors; ARBs, Angiotensin receptor blockers, NSAIDs, Nonsteroidal anti-inflammatory drugs.
