# Supplementary material for: Cartilage destruction in early rheumatoid arthritis patients correlates with CD21−/low double-negative B cells
Source: Arthritis Res Ther. 2024 Jan 15;26:23. doi: 10.1186/s13075-024-03264-2 (PMC10789032; doi:10.1186/s13075-024-03264-2)
Supplement: Supplementary file 3 — Additional file 3: Table S3. Comorbidities of eRA patients included in study. [file 13075_2024_3264_MOESM3_ESM.docx]

**Table S3. Comorbidities of eRA patients included in study**

| **Comorbidities** | **eRA**  **(N=59)** |
| --- | --- |
|  | *Number (%)* |
| None | 16 (27) |
| Cardiovascular disease | 17 (29) |
| Hypothyroidism | 6 (10) |
| Diabetes | 4 (7) |
| Asthma | 4 (7) |
| Osteoarthritis | 3 (5) |
| Previous cancer | 3 (5) |

Data are number of patients (%) for categorical data.
